# Effect of environmental noise and music on dexmedetomidine-induced sedation in dogs

**DOI:** 10.7717/peerj.3659

**Published:** 2017-07-31

**Authors:** Julia D. Albright, Reza M. Seddighi, Zenithson Ng, Xiaocun Sun, DJ Rezac

**Affiliations:** 1 Department of Small Animal Clinical Sciences, College of Veterinary Medicine, University of Tennessee—Knoxville, Knoxville, TN, United States of America; 2 Department of Large Animal Clinical Sciences, College of Veterinary Medicine, University of Tennessee—Knoxville, Knoxville, TN, United States of America; 3 Office of Information Technology, University of Tennessee—Knoxville, Knoxville, TN, United States of America; 4 Veterinary & Biomedical Research Center, Inc., Manhattan, KS, United States of America

**Keywords:** Noise, Dexmedetomidine, Canine, Classical music, Sedation

## Abstract

**Background:**

Previous studies in human patients suggest depth of sedation may be affected by environmental noise or music; however, related data in domestic animals is limited. The objective of the current study was to investigate the effect of noise and music on dexmedetomidine-induced (DM- 10 µg/kg, IM) sedation in 10 dogs.

**Methods:**

In a crossover design, post-DM injection dogs were immediately subjected to recorded human voices at either 55–60 decibel (dB) (Noise 1) or 80–85 dB (Noise 2); classical music at 45–50 dB (Music); or background noise of 40–45 dB (Control+). Control− included IM saline injection and exposure to 40–45 dB background noise. Sedation was assessed via monitoring spontaneous behavior and accelerometry (delta-g) throughout three 20-min evaluation periods: baseline, noise exposure, and post-treatment. Sedation was further assessed during two restraint tests at 30 min (R1) and 40 min (R2) post-injection. A mixed model for crossover design was used to determine the effect of noise exposure and time on either spontaneous behavior scores or delta-g. The restraint scores were analyzed using a two-way repeated measures ANOVA.

**Results:**

Spontaneous behavior scores indicated less sedation during Noise 2 compared to Control+ (*P* = 0.05). R2 restraint scores for all DM treatments except Noise 2 indicated significantly higher sedation than Control− [C+ (*P* = 0.003), M (*P* = 0.014) and N1 (*P* = 0.044)].

**Discussion:**

Results suggest that the quality of sedation is negatively impacted by high-intensity noise conditions (80–85 dB), but exposure to music did not improve sedation in this population of research dogs.

## Introduction

The acoustic environment of veterinary hospitals and its effects on patients has not been fully analyzed, although the impact of noise has been extensively scrutinized in human healthcare (Hasfeldt, Laerkner & Birkelund, 2010; Hsu et al., 2012; Konkani & Oakley, 2012). Nevertheless, noise stimulation in animals increases arousal and induce “fight or flight” behavioral and physiological responses through the autonomic and endocrine systems (Turner, Bauer & Rybak, 2007). The World Health Organization defines noise as “unwanted sound” and has identified hospital patients as a population particularly susceptible to the deleterious effects of noise (Berglund, Lindvall & Schwela, 1999). Heart rate, anxiety, and ventricular arrhythmias (Morrison et al., 2003), in addition to poor sleep quality, delayed wound healing, and diminished immune response have resulted in human patients being treated within loud hospital environments (Pope, 2010; Hsu et al., 2012). Also in human patients, it has been shown that obtaining a stable preoperative sedation under noisy conditions is significantly hindered and may necessitate the use of additional medications, potentially compromising the respiratory and cardiovascular stability of the patient (Rego, Watcha & White, 1997).

The decibel (dB) quantifies energy within sound waves and is the most common unit of measurement for sound intensity (Berglund, Lindvall & Schwela, 1999; Hsu et al., 2012). According to the Centre for Disease Control and Prevention, a normal human conversation is approximately 60 dB, a leaf blower at 15 m distance creates a 90 dB sound, and standing by a siren at close range is perceived as 120 dB (Centers for Disease Control and Prevention, 2015). Based on sound assessment studies conducted in intensive care units in veterinary hospitals, sound spikes above 80 dB were consistently detected (Fullagar et al., 2015).

In contrast to the negative effects of noise, perioperative use of slow-tempo music has received attention as a source of anxiolysis and analgesia in human surgical patients (Moris & Linos, 2013; Hole et al., 2015; Van der Heijden et al., 2015). Although the studies evaluating anxiolytic effects of music in veterinary patients are limited, the use of classical music has shown some potential in improving stress-related behaviors and physiologic parameters in both cats and dogs (Bowman et al., 2015; Mira et al., 2016).

As a common practice in veterinary medicine, patients often receive an intramuscular (IM) injection of sedative agents to achieve sedation, anxiolysis and increased compliance for performing minor procedures or establishing an intravenous access prior to general anesthesia. Dexmedetomidine (DM) is a highly selective alpha-2 adrenergic agonist used to achieve sedation and as a preanesthetic agent in veterinary patients (Granholm et al., 2007; Canfrán et al., 2016), although it may induce transient adverse cardiovascular effects that could be clinically detrimental in hemodynamically unstable patients or those with significant pre-existing diseases (Murrell & Hellebrekers, 2005). Beneficial effects of dexmedetomidine are mediated by activation of alpha-2 adrenergic receptors and a decrease in noradrenaline release and CNS excitation (Murrell & Hellebrekers, 2005; Granholm et al., 2007).

The objective of the present study was to determine the effects of noise with various intensities and classical music on the quality of DM-induced sedation in dogs. Sedation was assessed by scoring spontaneous behavior, evaluating movement through the use of an accelerometer and response to restraint. We hypothesized that dogs exposed to loud noise would be less sedated compared with those exposed to classical music and control animals with no extra noise intervention.

## Materials and Methods

### Animals

Participants were 10 healthy male purpose-bred beagles (8 neutered, 2 intact) with a mean ± SD weight of 12.4 ± 1.9 kg and 4.2 ± 1.3 years of age. All procedures were approved by the University of Tennessee Animal Care and Use Committee (Protocol No. 2376). The dogs were individually housed in indoor kennels and maintained on regular 12 hr/12 hr light/dark cycle with light conditions between 0600–1800 hr. Dogs had free access to water in kennels and were fed at 1700 daily during this study, although those receiving sedation were given their meal the following morning. This feeding schedule allowed a fasting period of 12 h prior to exposure to an experimental condition.

### Testing area

Testing occurred in an isolated room with 1.2 m thick concrete walls. The thick walls and empty anteroom connecting the testing room to the main building insulated the area from outside noises. The testing arena within the room was a circular enclosure (0.7 H × 1.5 L m) constructed of plastic panel walls and cotton/polyester blankets. The entire experimental sessions were video-recorded on each day using two cameras (Sony HDR-CX44; Sony Corp., Tokyo, Japan) placed on tripods (Sunpak 8001UT Tripod; ToCAD America Inc., Rockaway, NJ, USA) on opposite ends of the enclosure, approximately 1 m above the floor. The intensity of the noise was measured by placing a sound meter (UT352; Uni-Trend Technology, Hong Kong) in the center of the enclosure, approximately 0.5 m above the floor. The baseline, or background noise was determined to be 40–45 dB based on the average maximum and minimum readings from the sound meter recorded over a period of 5 min, at 3 time points between 0800–1800 h on the day prior to testing when no animal was present or intervention was underway. Testing occurred between the hours of 0800 and 1500. The room was maintained at a dim luminance of 6.6 cd/m^2^ and 20°C temperature throughout the experiment.

### Experimental protocol

#### Acclimation protocol

Acclimation occurred two days prior to the experiment by placing an accelerometer on the ventral portion of each dog’s collar (HeyrexVet, Heyrex, Limited). Dogs were then individually brought to the testing arena and allowed to investigate, receive attention, and eat canned food (Science Diet Adult Light, Hill’s Pet Nutrition, Topeka, KS, USA) from a rubber toy (Kong Classic; The Kong Company, Golden, CO, USA) for 30 min. To acclimatize the animals to the auditory stimuli, music and voice recordings, similar to those used in the experimental phase, were played during the last 10 min of the acclimation phase at approximately 5 dB above the background noise level. In addition, on intervening days between experiments, dogs were brought into the enclosure for 30 min and underwent the same acclimation protocol to reduce any aversion to the testing arena that may have developed on experiment days.

#### Treatment protocol

**Figure 1:**
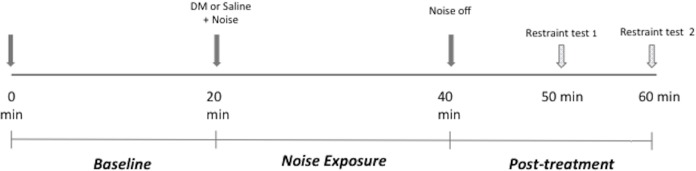
Experimental session timeline. DM, dexmedetomidine.

Using a Latin-square crossover design, each dog was assigned to be exposed to all of the treatment conditions with a minimum of 48 h between the treatments. The timeline of the experiment on each day is shown in Fig. 1. Each experiment was comprised of three 20 min periods for a total of 60 min. Briefly, on each experimental day, an activity monitor collar was fitted to the animal at least 1 h prior to testing. The animal was then placed in the enclosure for 20 min. During this period, referred to as *baseline period* in this report, the animal was only exposed to the premeasured background noise (40–45 dB) and was evaluated using accelerometry and spontaneous behavior scoring as described below (*Evaluation of sedation*). At the end of this period, the dog was given either DM (Dexdomitor, Zoetis) (10 μ g/kg, IM, epaxial muscle) or an equivalent volume of saline, according to the treatment assignment and was immediately subjected to one of the following five noise conditions for 20 min (*noise exposure period*):

- Negative control (C−): Saline injection and exposure to 40–45 dB background noise
- Positive control (C +): DM injection and exposure to 40–45 dB background noise
- Noise 1 (N1): DM injection and exposure to 55–60 dB recorded human voice
- Noise 2 (N2): DM injection and exposure to 80–85 dB recorded human voice
- Music (M): DM injection and exposure to 45–50 dB music.

All sounds were .wav sound files and played using an iPhone 6S (Apple Inc.) via two portable speakers (iHome iBT60; SDI Technologies, Rahway, NJ, USA) mounted to the tripods. At the onset of the noise exposure period, the dogs were immediately exposed to the noise or music at the predetermined dB level.

The music consisted of the initial 20 min of a commercial classical music marketed to produce a calming effect in dogs (Through a Dog’s Ear, Vol 1, BioAcoustic Research, Inc.). A dB of 45–50 was selected as the experimental music level after preliminary trials with different laboratory dogs exposed to the same music clips seemed the most relaxed at this dB level. N1 and N2 auditory stimuli were created by layering and staggering the recordings of two male and two female voices reading the same text, and looping the 90 sec clip for continuous play. Recordings were made using a Tascam DR-40 (TEAC America, Inc., Montebello, CA , USA) handheld 4-track recorder. A level of 55–60 dB was chosen for N1 based on estimated average dB level of the authors’ veterinary hospital anesthesia area over 5 min on three different typical medical procedure days. The N2 dB 80–85 level was selected as the highest experimental level (N2) because it has been documented in veterinary hospitals (Fullagar et al., 2015) but remains below the level considered dangerous to the hearing of humans (Berglund, Lindvall & Schwela, 1999; Centers for Disease Control and Prevention, 2015). To the authors’ knowledge, no data on dB levels at which dog hearing is damaged in dogs are available. Nevertheless, these safety guidelines were followed to minimize risk to the dogs and humans involved in the study.

After the noise exposure period, the applied noise or music playing was stopped and the animal was exposed only to the previous background noise for another 20 min (*post-treatment period*) during which the evaluation of sedation was continued.

#### Evaluation of sedation

The effect of each treatment on the animal was assessed using three methodologies; evaluating the spontaneous behavior, evaluating the body movements via accelerometry and response to a restraint test.

**Table 1:** Spontaneous behavior scoring description and rubric. The summed behavioral scores (body, head and eye) were averaged to determine a composite score for each evaluation period.

Behavior	Score	Description
Posture	4	Lateral recumbency, one shoulder and hip in full contact with floor, and/or 4 limbs extended
	3	Front limbs sternal, hip shifted laterally, hind limbs extended
	2	Sternal recumbency, hip and/or shoulder shifted laterally, limbs tucked under body, neck turned laterally
	1	Sternal recumbency, 4 limbs square
	0	Sitting
	−1	Standing motionless >3 s
	−2	Continuously walking
	−3	Circling, digging, pawing
Head	1	Head down
	0	Head up
Eyes	3	Sunken, unfocused
	2	Closed > 60 s
	1	Closed > 10 s <60 s
	0	Open > 10 s
	−1	Scanning, 3+ movements of pupil from center position/s

Animals’ spontaneous behavior was assessed every 5 min throughout the entire experiment, and an average score was calculated and analyzed for each evaluation period (baseline, noise exposure and post-treatment). The scoring system used was adapted from previously reported canine sedation scales (Girard et al., 2009; Hofmeister, Chandler & Read, 2010; Hopfensperger et al., 2013) with some modifications (Table 1). A single blinded observer (JA) performed the behavioral assessments using video recordings in a randomized order (arranged and coded by a non-observer) and played without sound. Both video camera angles were available for scoring but a single view was used at each assessment time point because the salient body postures or behaviors used for scoring were only visible from one view at any given time in the majority of trials. The summed behavioral scores (body, head and eye) were averaged to determine a composite score for each evaluation period. Possible scores ranged from 8 (least sedate) to −4 (most sedate).

For accelerometry, the activity monitor was placed on the collar to provide continuous data regarding total body movement as an additional indicator of treatment effect on animals’ behavior. Briefly, a tri-axial accelerometer captured dynamic body acceleration along three axes throughout the entire 60 min of the experimental session (Chen & Bassett Jr, 2005; Moreau et al., 2009). These raw vector magnitudes were transformed into gravity units (g) by proprietary software algorithms. Delta-g is defined as the absolute change in acceleration from the previous data point to the current and is a proxy for total movement during a predetermined time period. For this study, data were condensed into one-minute epochs, averaged, and analyzed for each evaluation period of the experiment. As a point of reference for this device, a beagle-sized dog would produce a delta-g of close to 0 when sleeping and approximately 200–250 delta-g at a continuous walk during a 1-min epoch (Table S1).

**Table 2:** Resistance scoring description and rubric. Scores were combined as one composite restraint score for each dog and each assessment point, restraint 1 (R1) and restraint 2 (R2).

Behavior	Score	Description
Restraint	1	No resistance (no struggling or appreciable muscle tone)
	0	Mild Resistance (no struggling or movement away, but muscle stiffening)
	−1	Strong resistance (struggle)
Post-restraint	2	No movement
	1	Ataxic standing
	0	Immediately stand or sit, calm
	−1	Escape attempts
Noise	2	No reaction
	1	Ears-only orient
	0	Head orients but no jump
	−1	Jump away

To simulate clinical conditions, restraint testing was performed during the post-treatment period at 30 min (Restraint 1- R1) and 40 min (Restraint 2- R2) after DM or saline injection (Fig. 1). Restraint testing was performed by a single blinded observer (JA) and at each time point consisted of encouraging the animal to walk to the investigator, restraint for a simulated cephalic catheter placement, and exposure to a sharp noise (a metal bowl being dropped from 0.5 m above the floor, at a distance of 1 m from the arena). The same blinded observer evaluated and scored the ability of the subject to walk to the observer, resistance to restraint, and reaction to sharp sound at R1 and R2 time points. Scoring was performed using a previously reported canine sedation scale (Girard et al., 2009; Hofmeister, Chandler & Read, 2010; Hopfensperger et al., 2013) with some modifications (Table 2). The scores for each of the phases of the restraint testing were combined and reported as one composite restraint score for R1 and R2 time points. Possible scores ranged from 5 (most sedate) to −3 (least sedate) for each time point.

#### Statistical analysis

Average scores for spontaneous behavior during evaluation periods and delta-g were analyzed using mixed models for crossover design to determine the effects of evaluation period and treatment, with subject as the random effect and treatment and evaluation period as repeated factors. For the delta-g analysis, post-treatment period data were analyzed separately and not included in the model with baseline and noise exposure periods because the restraint testing induced non-spontaneous activity during the post-treatment period. The restraint scores were analyzed using a two-way repeated measures ANOVA.

Ranked transformation was applied when data violated ANOVA assumptions such as non-normality and unequal variance. *Post hoc* multiple comparisons among evaluation period and treatment scores were conducted with Tukey’s adjustment. Statistical significance was identified at or below the level of 0.05. All analyses were conducted in SAS 9.4 TS1M3 (SAS Institute Inc.).

## Results

### Spontaneous behavior scores

**Figure 2:**
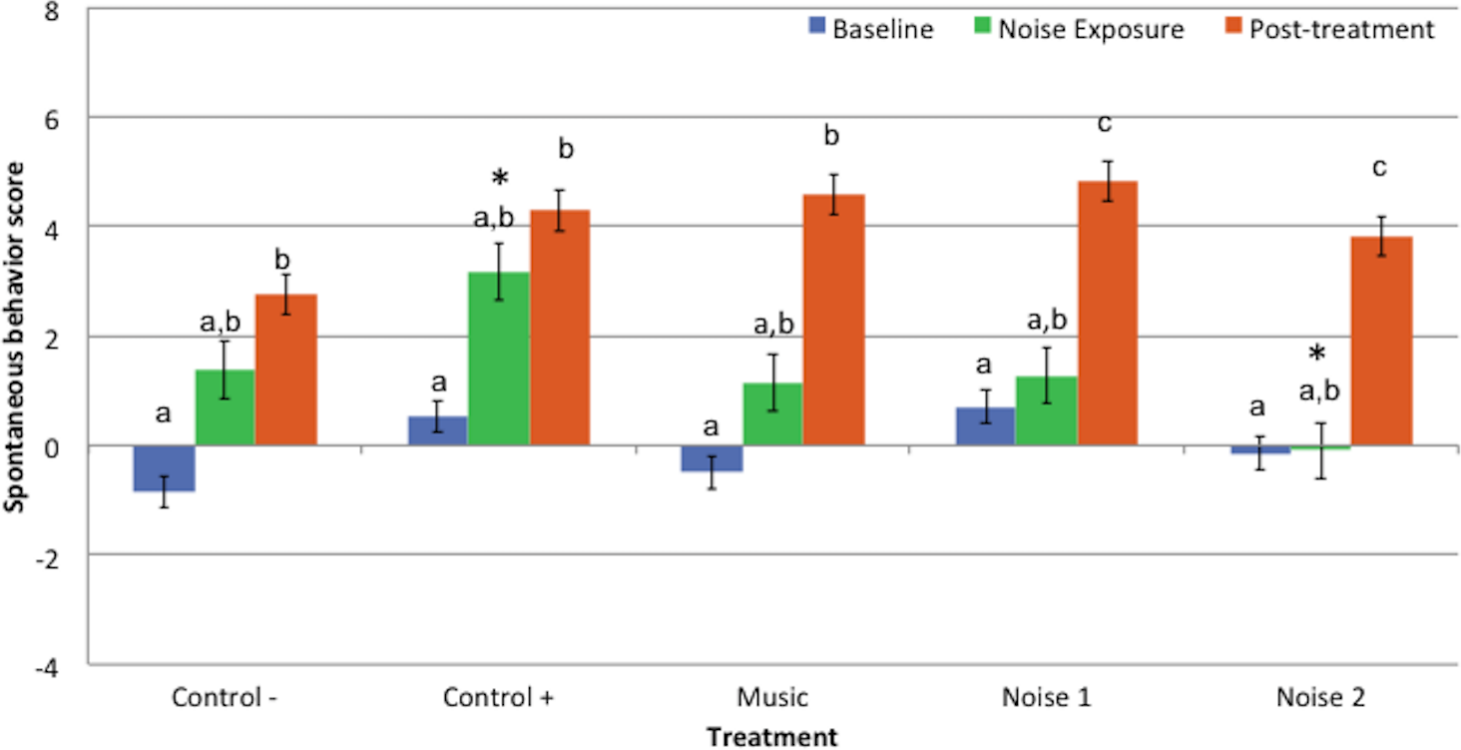
Mean spontaneous behavior scores ± standard error of the mean for 3 evaluation periods (baseline, noise exposure, post-treatment) in 5 treatments (Control−, Control+, Music, Noise 1, Noise 2) (*n* = 10 dogs). Higher scores reflected more sedation. Different letters indicate significant differences (*P* ≤ 0.05) within each treatment. * Indicates significant difference (*P* = 0.05) during noise exposure period between treatments Control+ and Noise 2.

Behavior scores during noise exposure period were similar to the baseline values for all treatments. Post-treatment scores were significantly higher than the baseline scores for all treatments [C− (*P* = 0.015), C + (*P* = 0.022), M (*P* = 0.001), N1 (*P* = 0.006), N2 (*P* = 0.011)]. Behavior scores during post-treatment periods for treatments N1 and N2 were higher than their respective noise exposure periods [N1 (*P* = 0.046) and N2 (*P* = 0.015)] but did not differ for any other treatment (Fig. 2).

Spontaneous behavior scores for baseline or post-treatment periods did not differ significantly among treatments. Behavior scores during noise exposure period in treatment N2 were lower than C + (*P* = 0.05) but did not differ significantly among other treatments (Fig. 2).

### Accelerometry (delta-g)

**Figure 3:**
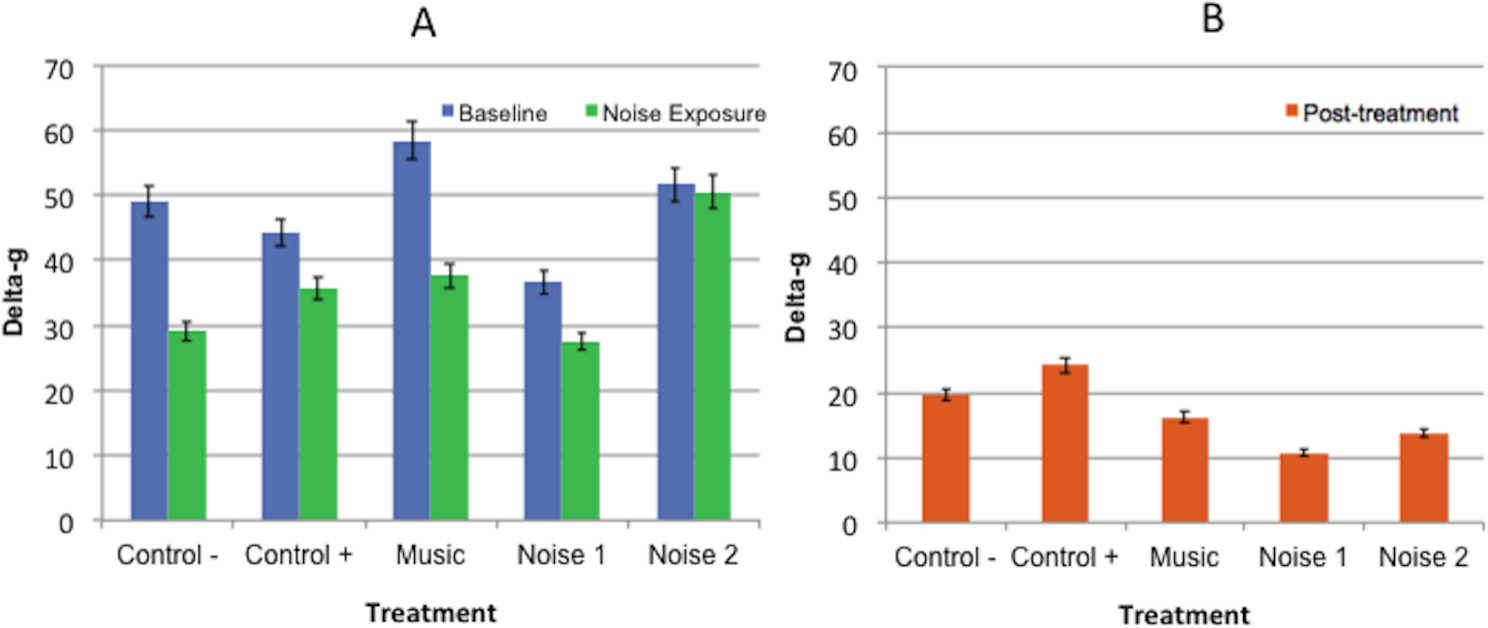
Mean delta-g values ± standard error of the mean for 3 experimental periods (baseline, noise exposure, post-treatment) in 5 noise treatments (Control−, Control+, Music, Noise 1, Noise 2) (*n* = 10 dogs). Higher scores reflected more movement. (A) No significant differences found in delta-g values collected during baseline evaluation and noise exposure periods within or among treatments. (B) No significant difference was found among post-treatment evaluation periods.

There were no significant differences in delta-g values for any of the periods among treatments or between baseline and noise exposure periods for any treatment (Fig. 3).

### Restraint test scores

**Figure 4:**
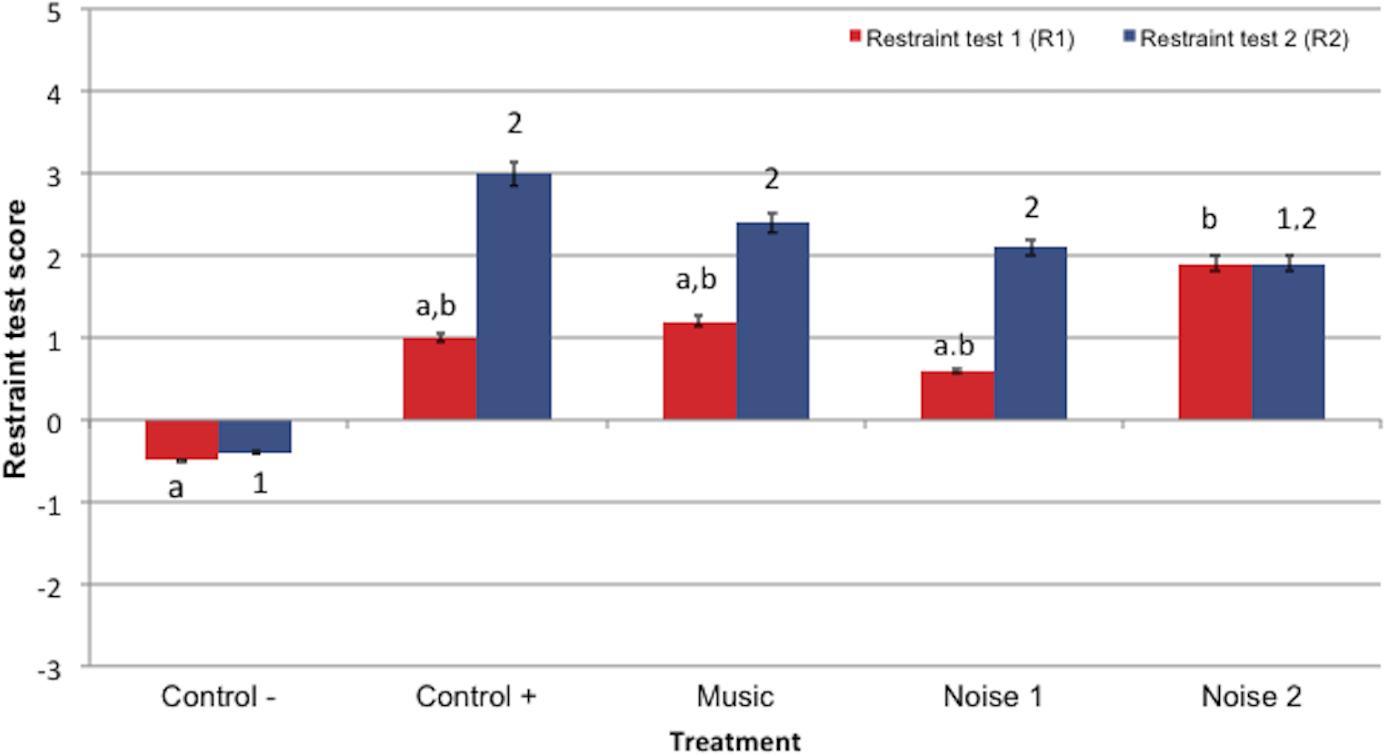
Mean restraint scores ± standard error of the mean for restraint test 1 (R1) and restraint test 2 (R2) in 5 noise treatments (Control−, Control+, Music, Noise 1, Noise 2) (*n* = 10 dogs). Higher scores reflected more sedation. Different letters indicate significant differences (*P* ≤ 0.05) at R1 time point treatments and different numbers indicated significant differences (*P* ≤ 0.05) R2 time points among treatments.

Restraint scores at R1 time point were significantly higher in treatment N2 compared to the Control− treatment (*P* = 0.012) (Fig. 4). There were no significant differences among R1 scores for other treatments. Restraint scores at the R2 time point did not significantly differ among treatments that received DM (C +, N1, N2 and M), but the R2 scores for the majority of DM treatments (except treatment N2) were greater than the saline (Control−) treatment - [C + (*P* = 0.003), M (*P* = 0.014) and N1 (*P* = 0.044)]). Restraint scores did not differ significantly between R1 and R2 within treatments.

## Discussion

The results of this study suggest that noise pollution can affect the quality of DM-induced sedation in dogs. Based on the spontaneous behavior scores, dogs receiving DM were more sedated while kept in an environment with less noise pollution [40–45 dB (Control+)] compared to more noisy conditions [80–85 dB (Noise 2)]. In other words, as was hypothesized, the depth of DM sedation may be significantly reduced if animals are exposed to a high intensity noise. The latter noise intensity approximates the sound of a heavy truck passing at 15 m (Centers for Disease Control and Prevention, 2015), and noise levels approaching this range have been reported in veterinary hospital treatment areas (Fullagar et al., 2015). It is thus reasonable to assume that sedation in veterinary patients may be compromised by such environmental noise pollution.

In the current study, neither spontaneous behavior nor delta-g scores during moderate intensity noise exposure treatments (50–60 dB, Noise 1 and Music) significantly differed from treatment Control+ (40–45 dB). Therefore, based on these results, this level of noise (40–60 dB), which approximates the decibel level of a typical human conversation (60 dB) (Centers for Disease Control and Prevention, 2015), may not significantly impact the level of sedation in dogs after receiving 10 µg/kg of DM, IM. However, the dogs in Control+ treatment appeared to be more sedated clinically during the noise exposure period compared to the same period for Noise 1 and Music treatments. Furthermore, spontaneous behavioral scores did not statistically differ during noise exposure for Control+ (DM) and Control− (saline) treatments.

A type II error due to low sample size may be one factor contributing to a lack of significant differences between the Control+ and Control−, and Control+ and moderate noise/music treatment assessments. Preliminary power analysis based previous sedation scores indicated 10 subjects would be sufficient; however, post-hoc analysis using these data concluded a minimum number of dogs needed to detect a difference with α of 0.05 and a power of 80% in spontaneous would be 13–17 for these specific sedation evaluation metrics. Alterations from previously published behavior and restraint scoring scales made in an attempt to increase the scale specificity for lighter sedation may account for the decrease in power.

Individual behavioral variation in response to noise stimuli may have also confounded the spontaneous behavior and delta-g results, particularly for the noise exposure period comparisons. As in other noise aversion studies (Landsberg et al., 2015; Gruen et al., 2015), some dogs showed active anxiety behaviors (pacing, panting, excessive self-directed licking) in response to the distressing noise stimuli, whereas others became very still but alert when exposed to the same noises. Including a trained observer score that accounts for active or passive behavior, or conducting a negative control for each of the noise conditions may have clarified these issues and strengthened the findings, although the latter would have exposed the dogs to additional potentially stressful treatments.

The sedentary behavior primarily displayed by the dogs, regardless of the treatment, also likely masked expected significant differences in spontaneous behavior scores and delta-g among the various noise and DM treatments. Habituation, or learning not to respond to a previously arousing stimulus, occurred throughout the trials, particularly the quiet periods, under dim lighting and otherwise constant environmental conditions despite several acclimation sessions in the room prior to and between experimental trials. Stiles et al. (2011) also found a reduction of some behaviors in dogs due to habituation after receiving a placebo injection; however, laboratory cats given an oral placebo did not have a decrease in activity between the first and second 30-min increments when assessed in their home cage environment (Orlando et al., 2015). The brighter lighting, presence of toys, detection of conspecifics and strong familiarity with the setting, as well as species-specific variance may explain the difference in initial activity levels between the studies.

Providing an additional acclimation period on the day of the experimental trials or extending experimental periods beyond 20 min may have decreased the impact of the habituation effect. Longer periods may have also provided a more appropriate duration for the DM treatment effect. Twenty minutes was chosen based on previous studies indicating that the onset of detectable sedative effects from 10 μ g/kg DM administered IM is approximately15 min, with peak effects around 30 min, and sedation assessments significantly higher than baseline for at least 60 min post-injection (Congdon et al., 2011). However, the semitendinosus muscle was the injection site in that study and Carter, Lewis & Beths (2013) demonstrated a slower onset of clinical sedation from DM and hydromorphone injected to the lumbar epaxial muscles when compared to the same drugs injected into the caudal thigh muscle. This was supported in the current study by higher restraint scores at 40 min compared to 30 min post-injection with DM. Longer trial periods or using caudal thigh as an injection site in the current study may have allowed the DM sedation to more fully take effect and demonstrated greater differences in sedation scores between DM and negative control treatments. Furthermore, a higher DM dose may have produced significant differences in the Control− and DM treatments, although this may have masked any effects among the noise treatment conditions.

In light of the issues regarding individual behavioral variation due to temperament and learning, restraint scores may be more specific indicators of sedation than spontaneous behavioral and activity assessments. At R2 time point (40 min after DM administration), as hypothesized, restraint scores for dogs in Control+, Noise 1, and Music, but not Noise 2 treatments, were significantly higher than saline Control− treatment scores. Performing an additional restraint test before the conclusion of the noise exposure period would have provided more clinically relevant data by assessing the effect noise during restraint. An unexpected finding was the increased level of sedation in dogs during the previous R1 time point (30 min post-DM injection) restraint testing for the loudest treatment (80–85 dB, Noise 2) compared to Control−, whereas the low and moderate noise intensity treatments (Control+, Noise 1, and Music) R1 restraint scores were not significantly higher than Control−. High arousal during the 80 dB noise exposure period may have led to increased fatigue in the dogs at the conclusion of the noise exposure when the restraint tests were performed.

The present study did not find any significant effect of classical music compared to the control treatment. This is in contrast to the reports claiming music may decrease anxiety and postoperative pain in people (Tharahirunchot, Tatiyapongpinij & Uerpairojkit, 2011; Moris & Linos, 2013; Hole et al., 2015; Van der Heijden et al., 2015), although other studies did not demonstrate any beneficial effect of music in decreasing the amount of drugs required to maintain an adequate plane of anesthesia (Kang et al., 2008; Ilkkaya et al., 2014). The assumed positive effect of music is mediated by activation of brain areas associated with rewards, as documented by functional magnetic resonance imaging (Salimpoor et al., 2013). For veterinary patients, slow-tempo classical music appears to have a calming effect on kenneled dogs (Wells, Graham & Hepper, 2002; Kogan, Schoenfeld-Tacher & Simon, 2012; Bowman et al., 2015), and in another study on anesthetized cats, it resulted in decreased blood pressure and heart rates when compared to other types of music (Mira et al., 2016). However, the proposed intrinsic “deactivating” and calming effects of slow-tempo music on the emotional state (Moris & Linos, 2013; Snowdon, Zimmermann & Altenmüller, 2015) in people may be affected by prior experience and exposure to the music (Walker, 1996). This may occur in animals as well and may have affected the results of the present study. Dogs used in this study were purposefully bred for research and had limited or no exposure to music. Therefore, performing a similar study using household pet dogs with prior music exposure may conclude different results in relation to the effect of music on behavior and sedation.

The results of this study may have been influenced by some other limitations. The findings from the entirely male subject pool, comprised of dogs available at the time of the study, may not generalize to female dogs. There are no known gender differences in sedation quality to the authors’ knowledge. Also, the dogs were only exposed to voice stimuli, not to the full range of sounds and frequencies that may induce a negative reactive reaction in patients undergoing preanesthetic procedures. Therefore, only conclusions regarding voice decibel levels should be drawn from these data.

Another possible limitation is the accelerometry device used in this study. The total activity of similar product (Whistle, Whistle Labs) was shown to strongly correlate with total activity from another monitor that has been validated in dogs (Actical, Animal Actical; Starr Life Sciences Corp., Oakmont, PA, USA) (Yashari, Duncan & Duerr, 2015). Those two products and the HeyRex use similar tri-axial accelerometer technology but the HeyRex device was chosen for use in this study because of both the affordability and the generation of a numerical measurement unit (delta-g) instead of graphic intensity bars. Nevertheless, the possibility that the data were not valid or delta-g was not specific enough to detect true differences among the periods or treatments cannot be ruled out.

Accelerometry in canine studies has been used primarily to assess activity patterns or pain over several days (Hansen et al., 2007; Guillot et al., 2011; Gerencsér et al., 2013). Orlando et al. (2015) provided short-term activity data using a different monitor (Actical) validated in cats after administration of trazodone, an antidepressant with sedating effects. Significant differences in activity were reported between medicated and placebo conditions for moderate and high doses of trazodone when data was assessed in 2-hour increments. However, only activity levels post-administration of the higher trazodone dose significantly differed from placebo when shorter 30-min increments were analyzed. Activity monitors may best employed when assessing activity over longer periods of time for low or moderate levels of sedation.

## Conclusion

The findings of this study suggest that less noisy conditions are optimal for achieving full sedation after DM administration, and noise intensity in veterinary hospitals, which commonly spikes up to 80 dB, likely has a negative impact on the quality of sedation. The findings of this study do not support the use of classical music to improve DM sedative effects. More studies on moderate intensity noise and various subject populations along with study design modification to strengthen findings are warranted.

##  Supplemental Information

10.7717/peerj.3659/supp-1 Supplemental Information 1 Delta g medium-sized dog walking gait estimate raw data

10.7717/peerj.3659/supp-2 Supplemental Information 2 Spontaneous behavior and restraint scores

10.7717/peerj.3659/supp-3 Supplemental Information 3 Accelerometer data
